# What is the role of pre-operative sperm DNA fragmentation index in microsurgical varicocelectomy success?

**DOI:** 10.3389/fsurg.2025.1703388

**Published:** 2026-01-12

**Authors:** Lihong Wang, Hui Jiang, Tao Jiang

**Affiliations:** 1 Department of Urology, Xuancheng People's Hospital, Xuancheng, China; 2 Department of Andrology, Peking University First Hospital, Beijing, China; 3 Department of Andrology and Sexual Medicine, The Second Hospital of Dalian Medical University, Dalian, China

**Keywords:** male infertility, microsurgical varicocelectomy, sperm, sperm DNA fragmentation index, varicocele

## Abstract

**Objective:**

This prospective study aimed to examine the effect of pre-operative sperm DFI on varicocelectomy success.

**Material and methods:**

A total of 51 infertile men with unilateral clinical VC who met the inclusion criteria and underwent microsurgical varicocelectomy were enrolled. As described in previous studies, in our study, more than 50% increase in total motile sperm count (TMSC) in post-operative semen analysis was defined as a significant improvement. However, at least a 100% increase was required for patients with a TMSC <5 million in the definition of recovery. The patients were separated as two groups as benefiting from the treatment and not.

**Results:**

Among the 31 patients who completed the 3-month follow-up, significant improvements were observed in sperm concentration, total sperm count, TMSC, abnormal sperm morphology, and DFI (*P* < 0.05). Post-operative sperm concentration, total sperm count, and TMSC increased, whereas abnormal sperm morphology and DFI decreased. Although mean DFI decreased across all VC grades, only grade III patients showed a statistically significant reduction (*P* < 0.05). Of the 31 patients, 16 exhibited semen quality improvement, while 15 did not. Preoperative sperm concentration, total sperm count, TMSC, and DFI significantly differed between the two groups (*P* < 0.05). A preoperative DFI threshold of ≥28.71% predicted post-operative semen improvement with 92.9% sensitivity and 50.0% specificity.

**Conclusions:**

Our study showed that high sperm DFI (≥28.71%) may be a useful pre-operative predictive tool in identifying men who benefit most from varicocelectomy in infertile patients with VC.

## Background

Varicocele (VC), characterized by abnormal retrograde blood flow in the pampiniform plexus leading to venous dilatation and tortuosity, represents a prevalent vascular disorder affecting approximately 15% of the general male population, with significantly higher incidence rates of 35%–44% in primary infertility and 45%–81% in secondary infertility cases (1, 2). While often asymptomatic, VC exerts detrimental effects on male fertility through multiple pathways including testicular hypoxia, elevated scrotal temperature, oxidative stress, and endocrine dysfunction, ultimately impairing spermatogenesis and testosterone production (1, 3, 4). The World Health Organization estimates that male factors contribute to 50% of infertility cases among reproductive-aged couples, with VC being one of the most common and treatable causes.

Contemporary management of palpable clinical VC primarily involves surgical intervention, with microsurgical varicocelectomy emerging as the gold standard due to its demonstrated superiority over traditional open and laparoscopic approaches in terms of postoperative complications, semen parameter improvement, and pregnancy rates (5, 6). While conventional semen analysis remains the cornerstone of male fertility assessment, its clinical utility is limited by significant intraindividual variability and the inability to predict fertilization potential in approximately 15% of infertile men with normal standard parameters. In contrast, evaluation of sperm DNA fragmentation (SDF) through established methodologies (TUNEL, SCSA, SCD, or Comet assay) provides more reliable prognostic information, as reflected by DNA fragmentation index (DFI) values (7, 8). These techniques demonstrate strong intermethod correlation (*r* = 0.59–0.9) and lower variability (<10%) compared to conventional semen parameters (28%–43%) (8).

Current sperm DFI thresholds stratify fertility potential as follows: ≤15% (normal), 15%–30% (intermediate), and ≥30% (impaired), with levels exceeding 40% associated with increased risks of pregnancy loss and congenital anomalies (9). A sperm DFI cutoff of 20% demonstrates optimal discriminative capacity between fertile and infertile populations (79% sensitivity, 86% specificity) (10). However, existing thresholds derive from general male populations rather than VC-specific cohorts. Although meta-analyses confirm consistent sperm DFI reduction following varicocelectomy across all VC grades, the absence of validated preoperative thresholds for surgical candidate selection represents a critical knowledge gap in clinical practice. This prospective study aims to systematically evaluate the effects of microscopic varicocelectomy on semen parameters and DFI in infertile VC patients, while establishing evidence-based thresholds to optimize patient selection and improve reproductive outcomes. Through comprehensive comparison of preoperative and 3-month post-operative semen kinetics, DFI, and morphological parameters, we seek to elucidate the relationship between sperm DFI and conventional semen indices, ultimately providing clinically relevant guidance for the management of VC-related infertility.

## Methods

### Study design and participants

This non-interventional prospective study was conducted at the Andrology Clinic of The Second Hospital of Dalian Medical University after obtaining approval from the Institutional Ethics Committee. From December 2023 to September 2024, we enrolled 51 infertile men with clinical VC who underwent microsurgical varicocelectomy, with 31 patients completing the 3-month postoperative follow-up. All participants provided written informed consent in accordance with the Declaration of Helsinki. The study adheres to CONSORT guidelines (Clinical trial number: PJ-KS-KY-2023-232).

#### Inclusion criteria

Age ≤40 years; Diagnosis of male infertility (failure to achieve pregnancy after 1 year of regular unprotected intercourse); Unilateral clinical VC confirmed by physical examination and color Doppler ultrasound; Meeting surgical indications for clinical VC (infertility with abnormal semen parameters or DFI > 15%, plus normal or treatable female fertility factors); Standard microsurgical subinguinal varicocelectomy performed (identifying and preserving testicular artery and lymphatics while ligating all internal spermatic and cremasteric veins); Willingness to undergo preoperative and 3-month postoperative semen analysis, DFI testing, and morphological evaluation; Agreement to avoid confounding medications/therapies during study period; Voluntary participation with signed informed consent.

#### Exclusion criteria

Age >40 years; Absence of male infertility; Subclinical or bilateral VC; Contraindications for VC surgery; Known factors affecting sperm DNA fragmentation (chronic diseases, genital infections, advanced age, unhealthy lifestyle, obesity, occupational/environmental exposures, medications, radiation/heat exposure); Inability to complete follow-up; Unwillingness to discontinue confounding treatments; Participation in other clinical trials within 3 months. In accordance with WHO criteria, obesity is defined as a BMI greater than 30.

#### Withdrawal criteria

Poor compliance (missed visits/tests or use of prohibited treatments); New confounding risk factors emerging during study; Post-operative complications or medical conditions precluding continuation; Patient dissatisfaction with treatment; Loss to follow-up; Withdrawal of consent.

#### Diagnostic criteria

Male infertility was defined as failure to conceive after 1 year of regular unprotected intercourse. Clinical VC was graded by physical examination: Grade I: Palpable only during Valsalva maneuver; Grade II: Palpable at rest, worsens with Valsalva; Grade III: Visible and palpable dilated veins at rest.

Color Doppler ultrasound confirmation was required for all cases, with physical examination findings taking precedence in case of discrepancy. The surgery was successful, pending confirmation of no reflux by postoperative Doppler. The presence and characteristics of varicocele were assessed using color Doppler ultrasonography (CDUS). Examinations were performed by a single experienced radiologist/urologist using a [Philips EPIQ 7] machine. Patients were examined in the supine and upright positions, both at rest and during a standardized Valsalva maneuver. A diagnosis of subclinical varicocele was made if retrograde venous flow (reflux) was detected only during the Valsalva maneuver. A clinical varicocele was defined by the presence of dilated pampiniform plexus veins (>2–3 mm in diameter in the supine position) and demonstrable reflux lasting ≥2 s during the Valsalva maneuver. The maximum venous diameter (MVD) and the duration of reflux were recorded for the largest vein identified.

### Laboratory methods

The study employed standardized laboratory protocols for comprehensive semen evaluation. Semen samples were collected after 2–7 days of abstinence and analyzed using computer-assisted semen analysis (CASA) to assess concentration, motility parameters, and total motile sperm count. Sperm DNA fragmentation was determined via sperm chromatin dispersion test, where at least 500 sperm per sample were classified based on halo patterns to calculate the DFI. Morphological assessment was performed on Diff-Quik stained smears under oil immersion microscopy, with strict quality control measures including standardized staining protocols and minimum counting requirements. All laboratory procedures followed WHO guidelines and were conducted by experienced technicians to ensure consistent and reliable results, with internal quality control demonstrating excellent reproducibility (intra-assay CV < 5%, inter-assay CV < 10% for key parameters).

Given the high variability of conventional semen analysis parameters, this study selected the Total motile sperm count (TMSC) – a relatively stable indicator derived by multiplying sperm count with motility rate – for comparison. According to relevant literature, the post-operative three-month improvement standard for semen quality was defined as: patients with pre-operative TMSC > 5 million showing over 50% increase in post-operative TMSC, and those with pre-operative TMSC <5 million achieving at least 100% increase (11–14).

### Statistical analysis

All statistical analyses were conducted using the Statistical Package for Social Sciences (SPSS) version 24.0 (SPSS Inc., Chicago, IL, USA) for Microsoft Windows. For normally distributed quantitative data, the mean ± standard deviation (Mean ± SD) is used; for non-normal distributions, the median {fourth percentile range [M(Q1, Q3)]} is employed. Statistical methods are selected based on data distribution characteristics: For normally distributed and homoskedastic data, independent samples t-test is applied to compare pre-and postoperative semen parameters. Non-parametric tests (Wilcoxon rank sum test) are used for non-normal distributions. Between-group comparisons of semen parameters across different VC classification groups employ one-way ANOVA. Spearman rank correlation analysis investigates the relationship between sperm DFI and semen parameters. A receiver operating characteristic (ROC) curve was constructed to determine the DFI prediction threshold for post-microsurgical varicocelectomy semen quality improvement, with AUC calculation and 95% confidence interval. All statistical tests were performed using two-tailed methods, with *P* < 0.05 as the threshold for statistically significant differences.

## Results

### Patient characteristics

This study enrolled 51 infertile patients with clinical left-sided VC who underwent microsurgical varicocelectomy, with a mean age of 28.8 ± 6.2 years. Based on clinical grading criteria, patients were stratified into three groups: grade I VC (*n* = 13), grade II VC (*n* = 10), and grade III VC (*n* = 28). No significant differences were observed in age or body mass index (BMI) among the three groups (*P* > 0.05).

Analysis of semen kinetic parameters revealed statistically significant differences among the groups in sperm concentration, total sperm count, and TMSC (*P* < 0.05). However, no significant intergroup differences were found in the proportion of morphologically abnormal sperm (*P* > 0.05). Notably, sperm DFI values differed significantly across the three VC severity groups (*P* < 0.05), as detailed in Table 1.

**Table 1:** Comparison of pre-operative basic data and semen parameters among different VC groups.

Variables	Total (*n* = 51)	Grade I (*n* = 13)	Grade II (*n* = 10)	Grade III (*n* = 28)	Statistic	*P*
Age (year), mean ± SD	28.8 ± 6.2	25.7 ± 7.4	29.1 ± 5.9	30.1 ± 5.5	*F* = 2.32	0.109
BMI (kg/m2), mean ± SD	24.84 ± 3.83	22.71 ± 3.09	26.06 ± 4.69	25.33 ± 3.55	*F* = 2.78	0.072
Sperm volume (mL), M (Q₁, Q₃)	4.00 (3.25, 4.55)	4.00 (3.40,5.00)	4.20 (3.20,4.38)	3.80 (3.18,4.40)	*χ*^2^ = 0.44	0.803
Sperm concentration (×106/mL), mean ± SD	53.66 ± 45.54	112.21 ± 52.67	55.15 ± 8.86	25.94 ± 11.48	*F* = 42.25	<0.001
PR (%), mean ± SD	29.22 ± 16.46	36.66 ± 14.30	32.87 ± 19.69	24.45 ± 15.05	*F* = 2.96	0.061
Total sperm count (×106), M (Q₁, Q₃)	130.60 (76.40, 323.52)	455.40 (396.04,509.56)	225.42 (164.15,249.10)	91.20 (62.67,118.50)	*χ*^2^ = 27.19	<.001
TMSC (×106), M (Q₁, Q₃)	38.84 (17.98, 119.16)	200.95 (163.68,275.57)	88.44 (31.22,109.56)	18.75 (13.93,38.96)	*χ*^2^ = 24.40	<.001
Abnormal proportion of sperm morphology (%), M (Q₁, Q₃)	99.04 (98.20, 99.73)	98.59 (97.63,99.18)	99.13 (98.12,99.73)	99.13 (98.74,100.00)	*χ*^2^ = 2.80	0.246
Sperm DFI (%), M (Q₁, Q₃)	37.03 (22.87, 52.11)	19.21 (17.10,19.65)	32.42 (25.68,39.70)	45.35 (36.67,62.46)	*χ*^2^ = 21.88	<.001

### Correlation analysis between sperm DFI and semen parameters

**Figure 1:**
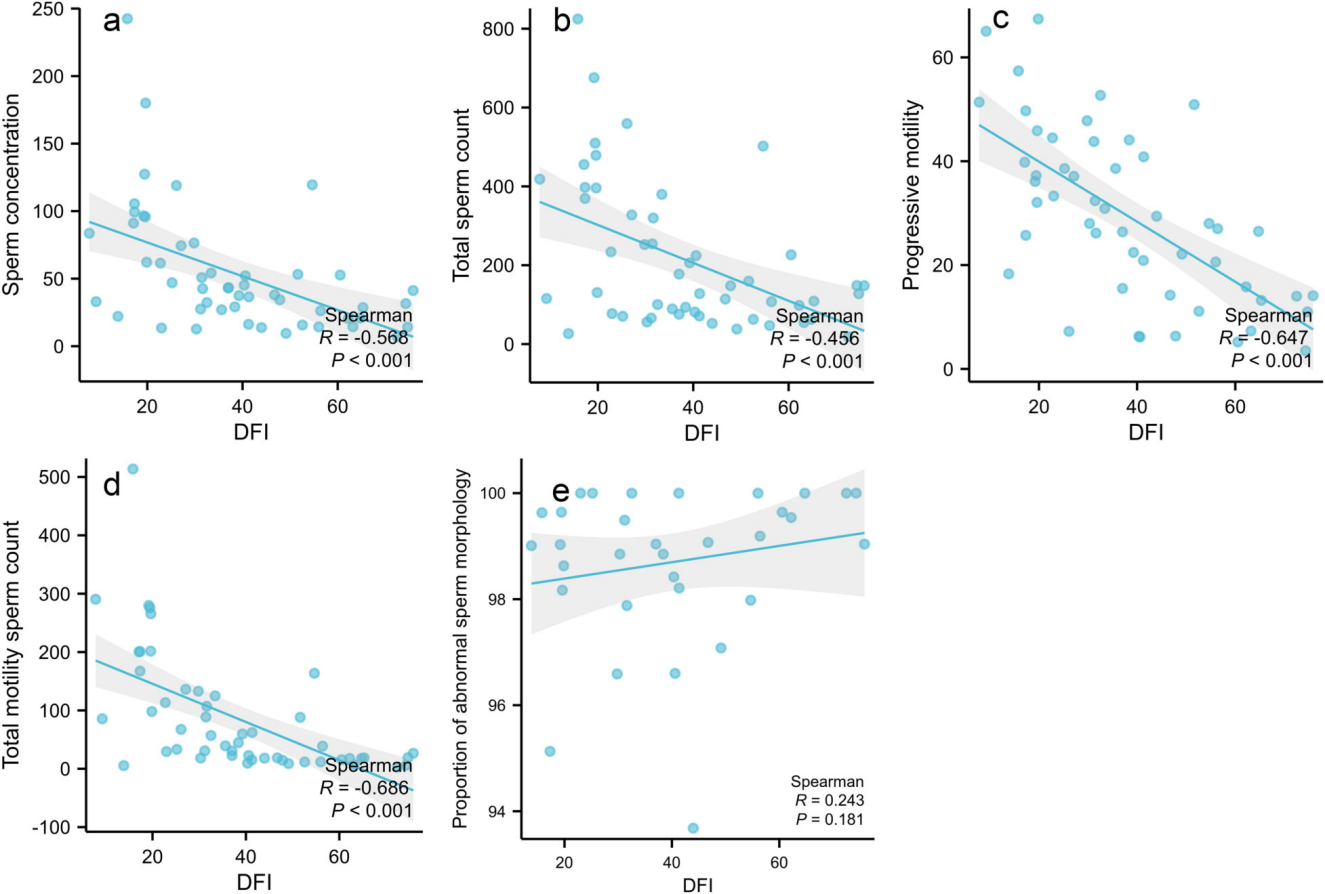
Correlation analysis between sperm DFI and semen parameters. **(a)** Sperm concentration; **(b)** Total sperm count; **(c)** Progressive motility; **(d)** Total motility sperm count; **(e)** Proportion of abnormal sperm morphology.

The DFI value of sperm showed negative correlations with sperm concentration (*r* = −0.568, *P* < 0.001), total sperm count (*r* = −0.456, *P* < 0.001), forward movement efficiency (*r* = −0.647, *P* < 0.001), and total motile sperm count (*r* = −0.686, *P* < 0.001). It was positively correlated with the proportion of morphologically abnormal sperm (*r* = 0.243, *P* = 0.181), as shown in Figure 1.

### Comparison of semen parameters before and 3 months after surgery

A total of 31 patients in this study completed follow-up examinations three months post-surgery. Significant differences (*P* < 0.05) were observed in sperm concentration, total sperm count, TMSC, proportion of morphologically abnormal sperm, and sperm DFI compared to preoperative levels. Post-surgery, sperm concentration, total sperm count, and TMSC showed improvement, while the proportion of morphologically abnormal sperm and sperm DFI decreased, as detailed in Table 2.

**Table 2:** Comparison of semen parameters before and 3 months after surgery.

Variables	Pre-operation (*n* = 31)	3 months after operation (*n* = 31)	Statistic	*P*
Sperm volume (mL), M (Q₁, Q₃)	26.16 (14.05, 35.20)	30.50 (18.80, 42.30)	*Z* = −0.98	0.326
Sperm concentration (×106/mL), mean ± SD	127.82 (74.15, 286.92)	194.10 (139.50, 316.75)	*Z* = −2.22	0.026
PR (%), mean ± SD	30.53 (16.64, 102.55)	64.05 (30.72, 144.29)	*Z* = −2.07	0.038
Total sperm count (×106), M (Q₁, Q₃)	99.34 (98.52, 100.00)	98.17 (97.29, 98.98)	*Z* = −2.17	0.030
TMSC (×106), M (Q₁, Q₃)	42.21 ± 17.55	30.24 ± 17.68	*t* = 2.50	0.016

### Comparison of pre-operative and 3 months post-operative sperm DFI in different graded VC patients

A total of 31 patients participated in this study and completed a three-month post-operative follow-up evaluation, with 6 cases classified as Grade VCI, 7 as Grade VCII, and 18 as Grade VCI. Three months after surgery, the average sperm DFI levels showed a decrease compared to pre-operative values. However, only Grade VCIII patients demonstrated a statistically significant difference in DFI between pre-operative and post-operative measurements (*P* < 0.05), indicating that the surgery had a more pronounced therapeutic effect on severe Grade VCI patients. See Table 3 for details.

**Table 3:** Comparison of pre-operative and 3 months post-operative sperm DFI in different graded VC patients.

Variables	Pre-operative DFI (%)	3 months post-operative DFI (%)	Statistic	*P*
Grade I (*n* = 6)	28.80 ± 17.74	26.05 ± 11.67	*t* = 0.23	0.827
Grade II (*n* = 7)	33.16 ± 13.47	26.57 ± 17.62	*t* = 0.79	0.447
Grade III (*n* = 18)	48.20 ± 15.43	31.92 ± 19.57	*t* = 2.51	0.019

### Predictive value of pre-operative sperm DFI on post-operative semen quality improvement and ROC curve analysis

Among 31 VC patients followed up three months post-operatively, 16 showed improved semen quality while 15 remained unchanged. Significant differences were observed between groups in pre-operative sperm concentration, total sperm count, TMSC, and sperm DFI (*P* < 0.05), indicating that sperm DFI has predictive value for postoperative semen quality improvement, as shown in Table 4.

**Table 4:** Comparison of pre-operative basic data and semen parameters between the group with improved semen quality and the group without improved semen quality 3 months after surgery.

Variables	Total (*n* = 31)	Non-improved group (*n* = 15)	Improvement group (*n* = 16)	Statistic	*P*
Sperm volume (mL), M (Q₁, Q₃)	4.20 ± 1.13	4.42 ± 1.35	3.99 ± 0.88	*t* = 1.06	0.296
Sperm concentration (×106/mL), mean ± SD	38.00 (26.60, 61.85)	54.20 (41.91, 82.75)	28.07 (15.74, 35.30)	*Z* = −2.91	0.004
PR (%), mean ± SD	28.01 ± 16.69	30.31 ± 17.86	25.86 ± 15.79	*t* = 0.74	0.467
Total sperm count (×106), M (Q₁, Q₃)	147.92 (93.60, 323.52)	233.80 (139.40, 388.55)	98.97 (65.10, 148.06)	*Z* = −2.86	0.004
TMSC (×106), M (Q₁, Q₃)	38.84 (18.05, 110.21)	98.08 (34.69, 130.56)	18.75 (15.02, 50.88)	*Z* = −2.43	0.015
Abnormal proportion of sperm morphology (%), M (Q₁, Q₃)	99.34 (98.69, 99.91)	99.12 (98.52, 99.56)	99.75 (98.90, 100.00)	*Z* = −1.21	0.225
Sperm DFI (%), M (Q₁, Q₃)	40.51 ± 17.31	33.47 ± 16.17	47.55 ± 15.94	*t* = −2.32	0.029

**Figure 2:**
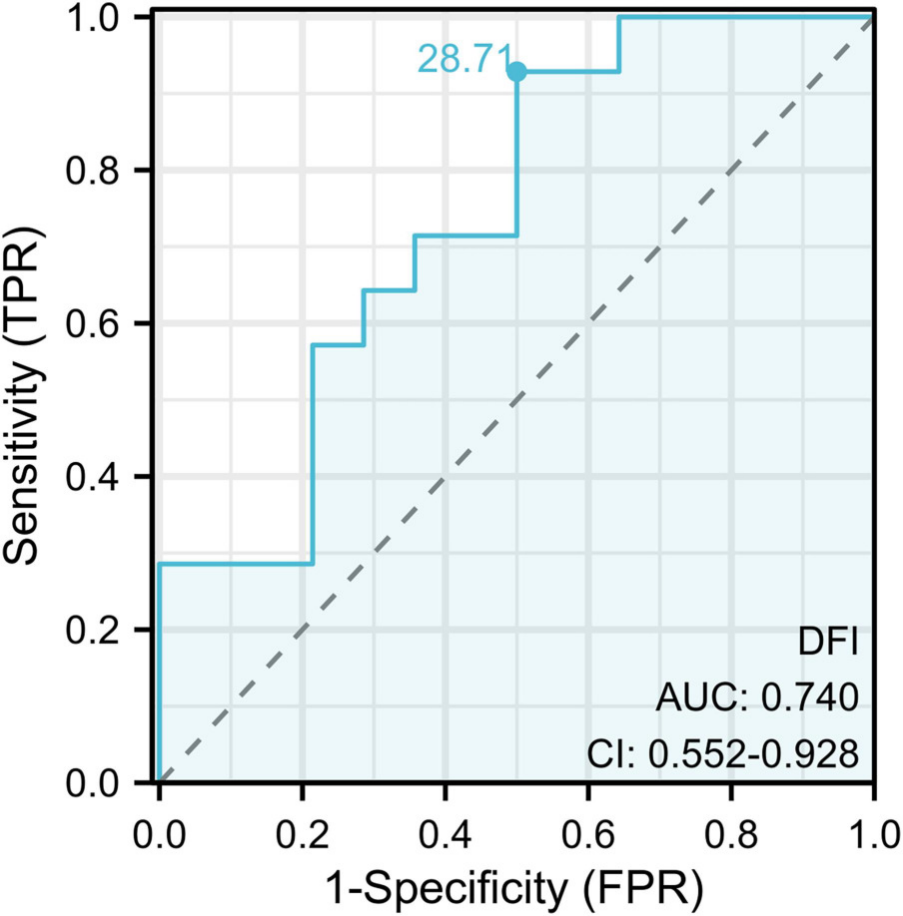
ROC curve of sperm DFI predicting postoperative semen quality improvement before surgery.

Based on post-operative semen quality improvement at 3 months, the ROC curve calculation determined a sperm DFI cutoff value of 28.71%, with an area under the curve (AUC) of 0.740 and a confidence interval of 0.552–0.928. The sensitivity was 92.9% and specificity 50.0% (*P* < 0.05) when sperm DFI ≥28.71%, indicating significant improvement in semen quality after microscopic varicocelectomy. This threshold serves as a crucial reference for predicting surgical efficacy, as shown in Figure 2.

## Discussion

VC, the most prevalent vascular pathology in the male reproductive system, is pathologically characterized by abnormal dilatation of the pampiniform venous plexus. This venous dilation typically results from valvular incompetence or anatomical defects in the spermatic veins, leading to impaired venous return and progressive vascular tortuosity. As the condition advances, it induces a spectrum of testicular dysfunction, including testicular volume reduction, seminiferous epithelium damage, and disruption of the hypothalamic-pituitary-gonadal axis, ultimately culminating in male infertility (15).

Clinical evidence demonstrates that VC causes multifaceted reproductive impairment: anatomically, it leads to testicular developmental abnormalities and localized discomfort; andrologically, it manifests as decreased sperm concentration, reduced progressive motility (PR), and increased morphological abnormalities. Epidemiological studies reveal VC prevalence rates of 14.8% in infertile men, 11.7% in the general adult male population, and strikingly 25.4% among men with abnormal semen parameters, underscoring its etiological significance in male infertility. Particularly, testicular spermatogenic dysfunction, as one of the most severe complications of VC, establishes it as a predominant cause of primary male infertility (1).

Although the detrimental effects of VC on male fertility are well-established, the precise pathophysiological mechanisms remain incompletely understood and are considered multifactorial. The principal mechanisms include: 1) thermal stress induced by scrotal hyperthermia; 2) toxic effects from metabolite reflux and accumulation; 3) inflammatory cascade and oxidative stress (OS) damage; 4) tissue hypoxia due to testicular microcirculatory impairment; and 5) heavy metal (e.g., cadmium) deposition. Furthermore, VC contributes to testicular dysfunction and infertility through additional pathways including mitochondrial dysfunction in spermatozoa, DNA integrity compromise, and induction of programmed cell death in testicular tissue (16–20).

Traditional male fertility assessment primarily relies on conventional semen parameters, which reflect basic semen characteristics but fail to reveal sperm function and reproductive potential. Studies indicate that approximately 15% of infertile men exhibit normal semen parameters (2). With technological advancements, the impact of DNA integrity on fertility has gained increasing attention. Research demonstrates that elevated sperm DFI correlates with decreased fertilization rates *in vitro*, increased rates of abnormal embryonic development, and potentially higher risks of cognitive impairment and chromosomal abnormalities in offspring (19). Factors contributing to DNA damage include unhealthy lifestyle habits (e.g., smoking, excessive alcohol consumption, sleep deprivation), radiation/chemotherapy, abnormal chromatin packaging, free radical attack, and environmental factors (20).

Conventional semen parameters serve as crucial indicators for assessing male fertility, reflecting overall sperm quality. Sperm DFI, as a marker of DNA integrity, provides insight into the health status of sperm DNA. In healthy males, the oxidative and antioxidant systems maintain dynamic equilibrium. Disruption of this balance leads to increased reactive oxygen species (ROS), triggering lipid peroxidation that damages sperm membranes and impairs motility. Oxidative stress also compromises sperm DNA integrity, causing strand breaks that reduce sperm quality, elevate DFI values, and ultimately affect fertilization outcomes. Such imbalances typically result from reproductive system abnormalities, with elevated DFI often accompanying reduced sperm viability and concentration. Additionally, aberrant sperm apoptosis and chromatin abnormalities can induce DNA damage, subsequently affecting sperm concentration, total count, and morphology. Therefore, sperm DFI values closely correlate with semen parameters and serve as important indicators for evaluating semen quality. Clinically, monitoring DFI values aids in assessing patients' semen status and determining infertility conditions.

Our findings demonstrate that as the clinical grade of VC increases in infertile patients, sperm concentration, total count, and TMSC progressively decline, while sperm DFI values show an upward trend. Preoperative sperm DFI negatively correlates with sperm concentration, total count, progressive motility, and TMSC, but positively correlates with the percentage of morphologically abnormal sperm. These findings collectively confirm the close relationship between sperm DFI and conventional semen parameters. Higher DFI values correspond to lower sperm viability and concentration, establishing DFI as a valuable reference index for assessing male semen quality.

Studies indicate that VC patients frequently exhibit abnormal sperm quality, with severity correlating with VC grade. VC induces testicular hyperthermia, circulatory impairment, increased vasoactive substances and oxygen free radicals, ultimately leading to deteriorated sperm quality and testicular damage (2). Our results show that preoperative semen parameters (except semen volume), including sperm concentration, total count, and motility, were significantly reduced, demonstrating VC's substantial impact on spermatogenesis and maturation. While traditional surgery, laparoscopy, and microsurgery all improve sperm concentration, motility, and morphology, microsurgical varicocelectomy demonstrates superior outcomes in enhancing sperm concentration and motility (5). Our study reveals that three months post-microsurgery, all VC patients showed significant improvements in sperm concentration, total count, and TMSC, along with marked reductions in abnormal sperm morphology and DFI, particularly in grade III patients where DFI reduction was most pronounced.

Given that clinical VC treatment significantly improves sperm DNA fragmentation, the Society for Translational Medicine 2017 clinical practice guidelines recommend DFI testing for infertile VC patients, especially grade II/III cases with normal semen parameters or grade I cases with borderline/abnormal parameters. Surgical intervention may be considered when DFI is elevated (21). Furthermore, as a key biomarker, sperm DFI holds multiple clinical applications in male reproductive medicine, including evaluation of VC treatment efficacy. Current research has extensively investigated DFI's predictive value for assisted reproductive technology (ART) outcomes, establishing threshold ranges for different detection methods: 4%–20% for TUNEL, 44%–56% for Comet assay, and 11.3%–30.3% (SCSA) and 17%–27.3% (SCD) respectively (22–27). Notably, significant variations exist between different DFI detection methods, and even for the same method, standardized diagnostic criteria remain lacking. These discrepancies likely stem from heterogeneity in study populations, sample sizes, and testing conditions. Therefore, reproductive centers should establish method-specific diagnostic thresholds based on their technical protocols and target populations (28). Currently, no consensus exists regarding surgical thresholds for infertile VC patients. Our results suggest that when using SCD, preoperative DFI≥28.71% predicts better postoperative semen quality improvement, providing valuable guidance for patient selection.

Despite the well-established benefits of VC repair in improving semen parameters and pregnancy rates, a subset of patients exhibit suboptimal or absent recovery postoperatively (29). Our findings are consistent with the literature. This heterogeneity in treatment outcomes underscores that VC is often one component within a broader spectrum of male infertility. The reasons for non-recovery are multifactorial and can be conceptualized across several domains. The most cited rationale for non-recovery is advanced, irreversible testicular damage sustained from prolonged venous hypertension, hyperthermia, and oxidative stress. Histological studies indicate that long-standing VC can lead to Sertoli cell-only pattern, hypospermatogenesis, or maturation arrest. A study demonstrated that a preoperative testicular biopsy showing severe spermatogenic dysfunction was a strong predictor of poor postoperative semen improvement (30). Similarly, the duration of infertility and the severity of preoperative oligozoospermia (especially sperm concentrations <5 million/mL) are consistently correlated with diminished recovery potential, suggesting a “point of no return” in the pathological cascade (31). VC is a significant source of elevated seminal OS and SDF. While repair generally reduces these parameters, a baseline severely elevated SDF may reflect damage that is not fully reversible. Smit et al. noted that while varicocelectomy significantly improved mean SDF, a portion of men with exceptionally high preoperative SDF remained with pathologic levels postoperatively, which may explain ongoing subfertility despite improved count and motility (32).

The traditional paradigm of VC repair has focused on the interruption of retrograde venous flow through ligation. However, a growing body of literature explores a more physiological approach aimed at reconstructing drainage. Some authors now advocate for the adjunctive use of microsurgical venous bypass—such as an internal spermatic to superficial epigastric vein anastomosis—in select complex cases or as a potential means to enhance outcomes (33). The underlying hypothesis is that beyond halting reflux, actively creating a low-resistance antegrade outflow tract may more effectively reduce intratesticular venous pressure and stagnation. This reconstructed drainage could theoretically improve the “washout” of testicular metabolic waste, reactive oxygen species, and catabolites, thereby ameliorating the biochemical milieu of spermatogenesis more comprehensively than ligation alone. While initial results from techniques like the one described by Papes et al. are promising, demonstrating resolution without recurrence in their series, robust comparative studies are needed to conclusively determine if this technically demanding addition translates into superior andrological outcomes, such as greater improvements in semen parameters or sperm DNA integrity, compared to high-level ligation techniques.

However, this study has certain limitations, such as a relatively small sample size and its single-center design, which may constrain the generalizability of the findings. Additionally, the postoperative follow-up period was relatively short. Future studies should analyze changes in sperm DFI at preoperative, 3-month, 6-month, and even longer postoperative timepoints to determine the optimal time to achieve maximum improvement and assess whether the therapeutic effects are sustained over time. Furthermore, investigations into the reasons for suboptimal postoperative DFI improvement are warranted. While this study primarily focused on semen quality improvement as the main outcome, future research should also examine whether reduced sperm DFI levels following surgery can ultimately enhance pregnancy success rates. The identified DFI threshold of ≥28.71% showed high sensitivity (92.9%) but moderate specificity (50.0%). The clinical implications of this moderate specificity may increase the risk of false positives and potential overtreatment. The lack of a control group (either non-surgical management or sham procedure) is a significant limitation that requires explicit acknowledgment. The potential variability in DFI measurement across different laboratories and testing methods, which could affect the applicability of their specific threshold value in other clinical settings. Meanwhile, we should acknowledge the potential confounders that might influence both DFI and surgical outcomes, such as lifestyle factors, age, duration of infertility, and female partner factors. Such studies would help better identify patients who are most likely to benefit from early surgical intervention, thereby preventing further deterioration of semen quality and fertility potential. These efforts would not only benefit infertile VC patients and their families but also contribute to improving population health, which represents the ultimate significance of this research.

## Conclusions

The sperm DFI of all graded VC patients decreased after surgery, especially in grade III patients. Microsurgical varicocelectomy significantly improved the semen quality of VC patients with high sperm DFI, especially when the pre-operative sperm DFI was ≥28.71%.

## Data Availability

The original contributions presented in the study are included in the article/Supplementary Material, further inquiries can be directed to the corresponding author.

## References

[1] Jensen CFS Østergren P Dupree JM Ohl DA Sønksen J Fode M. Varicocele and male infertility. Nat Rev Urol. (2017) 14(9):523–33. 10.1038/nrurol.2017.98 28675168

[2] Zavattaro M Ceruti C Motta G Allasia S Marinelli L Di Bisceglie C Treating varicocele in 2018: current knowledge and treatment options. J Endocrinol Invest. (2018) 41(12):1365–75. 10.1007/s40618-018-0952-7 30284221

[3] Agarwal A Sharma RK Desai NR Prabakaran S Tavares A Sabanegh E. Role of oxidative stress in pathogenesis of varicocele and infertility. Urology. (2009) 73(3):461–9. 10.1016/j.urology.2008.07.053 19167039

[4] Santana VP James ER Miranda-Furtado CL Souza MF Pompeu CP Esteves SC Differential DNA methylation pattern and sperm quality in men with varicocele. Fertil Steril. (2020) 114(4):770–8. 10.1016/j.fertnstert.2020.04.045 32709382

[5] Roque M Esteves SC. Effect of varicocele repair on sperm DNA fragmentation: a review. Int Urol Nephrol. (2018) 50(4):583–603. 10.1007/s11255-018-1839-4 29542060

[6] Tatem AJ Brannigan RE. The role of microsurgical varicocelectomy in treating male infertility. Transl Androl Urol. (2017) 6(4):722–9. 10.21037/tau.2017.07.16 28904905 PMC5583050

[7] Panner Selvam MK Ambar RF Agarwal A Henkel R. Etiologies of sperm DNA damage and its impact on male infertility. Andrologia. (2021) 53(1):e13706. 10.1111/and.13706 32559347

[8] Esteves SC Zini A Coward RM Evenson DP Gosálvez J Lewis SEM Sperm DNA fragmentation testing: summary evidence and clinical practice recommendations. Andrologia. (2021) 53(2):e13874. 10.1111/and.13874 33108829 PMC7988559

[9] Gill K Kups M Harasny P Machalowski T Grabowska M Lukaszuk M The negative impact of varicocele on basic semen parameters, sperm nuclear DNA dispersion and oxidation-reduction potential in semen. Int J Environ Res Public Health. (2021) 18(11):5977. 10.3390/ijerph18115977 34199549 PMC8199719

[10] Santi D Spaggiari G Simoni M. Sperm DNA fragmentation index as a promising predictive tool for male infertility diagnosis and treatment management - meta-analyses. Reprod Biomed Online. (2018) 37(3):315–26. 10.1016/j.rbmo.2018.06.023 30314886

[11] Duran MB Kizilkan Y Senel S Yikilmaz TN Toksoz S. Can preoperative inflammatory markers predict the success of varicocelectomy? Andrologia. (2022) 54(9):e14514. 10.1111/and.14514 35753707

[12] Ok F Erdogan O Durmus E. Can preoperative gonadotropin and testosterone levels predict the success of varicocelectomy? Andrologia. (2020) 52(11):e13887. 10.1111/and.13887 33125763

[13] Erdogan O Ok F. The effect of systemic inflammatory index and systemic inflammatory response index on success of varicoselectomy. Urologia. (2024) 91(1):170–5. 10.1177/03915603231192739 37632401

[14] Ates E Ucar M Keskin MZ Gokce A. Preoperative neutrophil-to-lymphocyte ratio as a new prognostic predictor after microsurgical subinguinal varicocelectomy. Andrologia. (2019) 51(2):e13188. 10.1111/and.13188 30397905

[15] Hassanin AM Ahmed HH Kaddah AN. A global view of the pathophysiology of varicocele. Andrology. (2018) 6(5):654–61. 10.1111/andr.12511 29978951

[16] Krzyściak W Kózka M. Generation of reactive oxygen species by a sufficient, insufficient and varicose vein wall. Acta Biochim Pol. (2011) 58(1):89–94. 10.18388/abp.2011_2290 21383993

[17] Rao M Zhao XL Yang J Hu SF Le H Xia W Effect of transient scrotal hyperthermia on sperm parameters, seminal plasma biochemical markers, and oxidative stress in men. Asian J Androl. (2015) 17(4):668–75. 10.4103/1008-682X.146967 25652627 PMC4492061

[18] Jeremias JT Belardin LB Okada FK Antoniassi MP Fraietta R Bertolla RP Oxidative origin of sperm DNA fragmentation in the adult varicocele. Int Braz J Urol. (2021) 47(2):275–83. 10.1590/s1677-5538.ibju.2019.0827 33146981 PMC7857753

[19] Abdelbaki SA Sabry JH Al-Adl AM Sabry HH. The impact of coexisting sperm DNA fragmentation and seminal oxidative stress on the outcome of varicocelectomy in infertile patients: a prospective controlled study. Arab J Urol. (2017) 15(2):131–9. 10.1016/j.aju.2017.03.002 29071142 PMC5653613

[20] Dada R. Sperm DNA damage diagnostics: when and why. Transl Androl Urol. (2017) 6(Suppl 4):S691–4. 10.21037/tau.2017.05.26 29082201 PMC5643659

[21] Agarwal A Cho CL Majzoub A Esteves SC. The society for translational medicine: clinical practice guidelines for sperm DNA fragmentation testing in male infertility. Transl Androl Urol. (2017) 6(Suppl 4):S720–33. 10.21037/tau.2017.08.06 29082206 PMC5643607

[22] Tandara M Bajić A Tandara L Bilić-Zulle L Šunj M Kozina V Sperm DNA integrity testing: big halo is a good predictor of embryo quality and pregnancy after conventional IVF. Andrology. (2014) 2(5):678–86. 10.1111/j.2047-2927.2014.00234.x 24947544

[23] Esbert M Pacheco A Vidal F Florensa M Riqueros M Ballesteros A Impact of sperm DNA fragmentation on the outcome of IVF with own or donated oocytes. Reprod Biomed Online. (2011) 23(6):704–10. 10.1016/j.rbmo.2011.07.010 22019617

[24] Osman A Alsomait H Seshadri S El-Toukhy T Khalaf Y. The effect of sperm DNA fragmentation on live birth rate after IVF or ICSI: a systematic review and meta-analysis. Reprod Biomed Online. (2015) 30(2):120–7. 10.1016/j.rbmo.2014.10.018 25530036

[25] Cissen M Wely MV Scholten I Mansell S Bruin JP Mol BW Measuring sperm DNA fragmentation and clinical outcomes of medically assisted reproduction: a systematic review and meta-analysis. PLoS One. (2016) 11(11):e0165125. 10.1371/journal.pone.0165125 27832085 PMC5104467

[26] Simon L Castillo J Oliva R Lewis SE. Relationships between human sperm protamines, DNA damage and assisted reproduction outcomes. Reprod Biomed Online. (2011) 23(6):724–34. 10.1016/j.rbmo.2011.08.010 22036908

[27] Jin J Pan C Fei Q Ni W Yang X Zhang L Effect of sperm DNA fragmentation on the clinical outcomes for *in vitro* fertilization and intracytoplasmic sperm injection in women with different ovarian reserves. Fertil Steril. (2015) 103(4):910–6. 10.1016/j.fertnstert.2015.01.014 25747135

[28] Vandekerckhove FW De Croo I Gerris J Vanden Abbeel E De Sutter P. Sperm chromatin dispersion test before sperm preparation is predictive of clinical pregnancy in cases of unexplained infertility treated with intrauterine insemination and induction with clomiphene citrate. Front Med (Lausanne). (2016) 3:63. 10.3389/fmed.2016.00063 27933295 PMC5120098

[29] Baazeem A Belzile E Ciampi A Dohle G Jarvi K Salonia A Varicocele and male factor infertility treatment: a new meta-analysis and review of the role of varicocele repair. Eur Urol. (2011) 60(4):796–808. 10.1016/j.eururo.2011.06.018 21733620

[30] Chiba K Ramasamy R Lamb DJ Lipshultz LI. The varicocele: diagnostic dilemmas, therapeutic challenges and future perspectives. Asian J Androl. (2016) 18(2):276–81. 10.4103/1008-682X.167724 26698233 PMC4770499

[31] Samplaski MK Jarvi KA. Prognostic factors for a favorable outcome after varicocele repair in adolescents and adults. Asian J Androl. (2016) 18(2):217–21. 10.4103/1008-682X.169558 26732108 PMC4770489

[32] Smit M Romijn JC Wildhagen MF Veldhoven JL Weber RF Dohle GR. Decreased sperm DNA fragmentation after surgical varicocelectomy is associated with increased pregnancy rate. J Urol. (2010) 183(1):270–4. 10.1016/j.juro.2009.08.161 19913801

[33] Papes D Cavar S Sabolic I Pasini M Jurca I Antabak A Internal spermatic vein to superficial epigastric vein microsurgical bypass in varicocele treatment. Eur J Pediatr Surg. (2023) 33(2):138–43. 10.1055/s-0042-1750053 36104092

