# Supplementary material for: What is the role of pre-operative sperm DNA fragmentation index in microsurgical varicocelectomy success?
Source: Front Surg. 2026 Jan 12;12:1703388. doi: 10.3389/fsurg.2025.1703388 (PMC12868975; doi:10.3389/fsurg.2025.1703388)
Supplement: Supplementary file 1 [file Table1.docx]

*The reason why each specific patients was lost*

Due to loss to follow-up or withdrawal of some patients postoperatively, there is a gap in the complete dataset, as shown in Supplementary Table.

**Supplementary Table.** Detailing reasons for loss to follow-up for 20 patients

| **Patient ID** | **Primary Reason for Loss to Follow-up** | **Efforts Made to Retain Participant** |
| --- | --- | --- |
| P-01 | Relocation: Moved to another province due to job transfer. | Suggested completing tests at a local clinic. |
| P-02 | Lost to Contact: All phone numbers disconnected; address invalid. | ≥10 attempts via phone, text, registered mail, and emergency contacts. |
| P-03 | Voluntary Withdrawal (Study-Unrelated): Cited "personal time constraints." | Explained study importance; offered more flexible scheduling; declined. |
| P-04 | Lost to Contact (Unknown): Ceased communication abruptly for unknown reasons. | >3 contact attempts per protocol with no response. |
| P-05 | Adverse Event (Study-Related): Persistent mild discomfort led to loss of confidence in the study. | PI communicated to explain and address concerns; offered additional counseling. |
| P-06 | Death (Non-Study): Unrelated fatal traffic accident. | Information confirmed via public obituary; condolences sent to family. |
| P-07 | Dissatisfaction with Efficacy: Felt personal recovery was unsatisfactory and refused further participation. | Conducted clinical assessment; explained individual variability; encouraged continued monitoring. |
| P-08 | Relocation (International): Emigrated overseas with family. | Attempted video follow-up & online questionnaires; failed due to time zone/technical issues. |
| P-09 | Loss of Interest / Perceived Burden: Found follow-up procedures too cumbersome. | Offered simplified next visit & travel reimbursement; declined. |
| P-10 | Lost to Contact (Presumed Number Change): Phone number out of service; address incorrect. | Attempted to locate via community center; unsuccessful. |
| P-11 | Lost to Contact (Presumed Number Change): Phone number out of service; address incorrect. | Attempted to locate via community center; unsuccessful. |
| P-12 | Economic / Transportation Barriers: Unable to afford travel costs to the site. | Attempted switch to phone follow-up; calls unanswered. |
| P-13 | Voluntary Withdrawal (Reason Unspecified): Simply stated "do not wish to continue." | Attempted to elicit specific reason for study improvement; patient declined to specify. |
| P-14 | Lost to Contact (Unknown): Ceased communication abruptly for unknown reasons. | >3 contact attempts per protocol with no response. |
| P-15 | Privacy Concerns: Worried about personal health data security. | Detailed data encryption and confidentiality procedures; concerns not alleviated. |
| P-16 | Voluntary Withdrawal (Reason Unspecified): Simply stated "do not wish to continue." | Attempted to elicit specific reason for study improvement; patient declined to specify. |
| P-17 | Caregiver Burden: Primary caregiver fell ill, requiring patient to provide home care. | Proposed home visit; not feasible due to long distance (different city). |
| P-18 | Misunderstanding: Patient mistook postoperative check-up as the end of study participation. | Contacted promptly to clarify; patient unwilling to re-engage. |
| P-19 | Dissatisfaction with Staff: Unhappy with attitude of a research staff member during a visit. | Study coordinator apologized and offered different staff; patient refused. |
| P-20 | Voluntary Withdrawal (Reason Unspecified): Simply stated "do not wish to continue." | Attempted to elicit specific reason for study improvement; patient declined to specify. |
